# 16q24.3 Microduplication in a Patient With Developmental Delay, Intellectual Disability, Short Stature, and Nonspecific Dysmorphic Features: Case Report and Review of the Literature

**DOI:** 10.3389/fped.2020.00390

**Published:** 2020-07-15

**Authors:** Simona Bucerzan, Diana Miclea, Cecilia Lazea, Carmen Asavoaie, Andrea Kulcsar, Paula Grigorescu-Sido

**Affiliations:** ^1^Iuliu Haţieganu University of Medicine and Pharmacy, Cluj-Napoca, Romania; ^2^Children's Emergency Clinical Hospital Cluj-Napoca, Cluj-Napoca, Romania

**Keywords:** developmental delay, short stature, 16q24.3 duplication, osteoporosis, dysmorphic features

## Abstract

We describe the case of a seven-year-old female patient who presented in our service with severe developmental delay, intellectual disability, facial dysmorphism, and femur fracture, observed in the context of very low bone mineral density. Array-based single nucleotide polymorphism (SNP array) analysis identified a 113 kb duplication involving the morbid OMIM genes: *ANKRD11* (exon1), *RPL13*, and *PGN* genes. ANKRD11 deletions are frequently described in association with KBG syndrome, the duplications being less frequent (one case described before). The exome sequencing was negative for pathogenic variants or of uncertain significance in genes possibly associated with this phenotype. The patient presented subtle signs of KBG syndrome. It is known that the phenotype of KBG syndrome has a wide clinical spectrum, this syndrome being often underdiagnosed due to overlapping features with other conditions, also characterized by multiple congenital anomalies and intellectual disability. The particularity of this case is represented by the very low bone mineral density in a patient with 16q24.3 duplication. ANKRD11 haploinsufficiency is known to be associated with skeletal involvement, such as short stature, or delayed bone age. An effect on bone density has been observed only in experimental studies on mice with induced missense mutations in the *ANKRD11* gene. This CNV also involved the duplication of the very conserved *RPL13* gene, which could have a role for the skeletal phenotype of this patient, knowing the high level of gene expression in bone tissue and also the association with spondyloepimetaphyseal dysplasia Isidor Toutain type, in case of splicing mutations.

## Background

The copy number variants (CNVs) involving 16q24.3 region are often associated with KBG syndrome (OMIM 148050), which is a rare genetic condition with autosomal dominant inheritance, firstly described by Hermann in 1975 (1). Initially, this disorder was considered to be very rare, now we know that it is probably underdiagnosed due to milder form of disease, the studies on patients with developmental disability demonstrating that this genetic condition is a frequent etiology for these disorders (2, 3).

KBG syndrome is often caused by heterozygous mutations in the *ANKRD11* gene (ankyrin repeat domain–containing protein 11) or chromosomal microdeletion of 16q24.3 (3).

The *ANKRD11* gene codes for a chromatin regulator and controls histone acetylation. The gene product is an inhibitor of ligand-dependent transcriptional activation that plays an important role in the proliferation and development of cortical neural precursors and neural plasticity and may be also involved in normal bone development (4). *ANKRD11* appears to be a major gene associated with neurodevelopmental delay (2, 3).

Clinical manifestations of KBG syndrome include distinctive craniofacial features (triangular face, brachycephaly, hypertelorism, wide eyebrows, synophrys, prominent nasal bridge, elongated philtrum, thin upper lip, and macrodontia), short stature, skeletal abnormalities, and neurological involvement, with developmental delay, intellectual disability, and seizures (5). There are several recommendations to use clinical diagnostic criteria for KBG syndrome to facilitate accurate recognition of this condition (6–8). However, the clinical picture is not always very evocative for this disorder, Low et al. showing in a study on 32 patients with KBG syndrome that less than a half of these patients presented suggestive clinical features, the others having a more subtle clinical features, the diagnosis being established only after exome testing (8). They observed that the most common findings in KBG syndrome were not specific features, such as: developmental delay, speech delay, learning difficulties, or behavioral problems (8). Taking into account these data, Low et al. recommended using the major criteria (macrodontia or gestalt of KBG syndrome; height below 10th centile; recurrent otitis media or hearing loss; 1st degree relative with KBG syndrome) and the minor criteria (hand anomaly; seizures; cryptorchidism; feeding problems; palate abnormalities; formal diagnosis of autism; large anterior fontanelle, or delayed closures), the diagnosis being established if the patient present developmental delay/learning difficulties, speech delay, or significative behavioral issues with at least two major criteria or one major criteria and two minor criteria (8).

## Case Presentation

### Clinical Report

The patient, a seven-year-old girl, presented to the Department of Genetic Diseases of the Emergency Hospital for Children in Cluj-Napoca, Romania, due to a severe developmental delay, craniofacial abnormalities and femur fracture caused by a minor trauma.

The patient was born at 35 weeks of gestation by spontaneous vaginal delivery, after a pregnancy complicated by a double nuchal cord, birth asphyxia and postnatal intraventricular hemorrhage. At birth, the weight was 1,960 g (−0.56 *SD*), length 44 cm (−0.32 *SD*), and Apgar score 7/7 (1′/5′). During the pregnancy, the mother denied the possible exposal to toxic substances (i.e., alcohol or drugs), known infectious pathogens or other harmful environmental factors.

The patient presented with significant global developmental delay, first noticed at 6–12 months of age in the following areas: gross and fine motor skills (delay for all motor skills, with walking achieved at two years and four months), cognitive and social skills (diagnosed as autism spectrum disorder), speech and language (at the age of seven, she had a limited vocabulary of 10–15 words, could not build simple sentences and also to imitate familiar sounds and to understand simple instructions).

The patient had a first epileptic episode (generalized seizures), without a fever context, at 2½ years and has been treated with valproic acid, with no further seizures to date. No significant family history of congenital anomalies, intellectual disability, or other neuropsychiatric abnormalities were mentioned.

During the first admission to our department, we noticed microcephaly (head circumference 46.2 cm, −2.82 *SD*), short stature (height 101.7 cm, −3.97 *SD*), weight deficit (body mass index 12.1 kg/m^2^, −2.61 *SD*). Auxologic parameters were calculated according to the growth charts published by the Romanian Society of Endocrinology for the Romanian population (birth parameters and height) and WHO Charts (head circumference and body mass index) (9, 10).

The patient presented with facial dysmorphism, featuring low frontal, and posterior hairlines, prominent anteverted ears, convergent strabismus, blue sclerae, telecanthus, broad nasal bridge with bulbous nasal tip, macrodontia of the upper central incisors, long philtrum, and thin upper lip, as well as short neck and bilateral clinodactyly of the fifth finger.

The patient also presented intellectual disability (IQ of 30), global developmental delay, autism spectrum disorder, and muscular hypotonia.

The diagnosis of intellectual deficiency was based on an IQ (for children older than six years) evaluated by the WISC-IV test (Wechsler Intelligence Scale for Children).

Bone age estimated using the Greulich–Pyle method, was concordant with chronological age. Hand radiography evidenced an osteoporotic aspect of this region. Echocardiography showed a bicuspid aortic valve. Abdominal ultrasounds did not reveal pathologic changes. MRI investigation did not show malformations or sequels indicating possible effects of perinatal asphyxia.

Laboratory tests showed moderate increased thyroid-stimulating hormone levels of 9.58 μIU/mL (normal range 0.58–5.2), associated with normal free thyroxine 1.09 ng/dL (normal range 0.8 – 1.7), interpreted as subclinical hypothyroidism. The patient was receiving treatment with L-thyroxin, 25 mcg/kg per day.

One month before admission to our department, the patient presented a transverse fracture across the proximal left femur that occurred due to a minor trauma (after falling on the same level).

Considering the clinical features, bone densitometry was performed, to exclude a pathological skeletal background. Bone mineral density values were significantly lower than the normal limits for her age and sex: left femur, with Z-score of −8.4; right femur with Z-score of −5.2 and L1–L4 region, with Z-score of −2.0. Before treatment, serum calcium, phosphorus, parathyroid hormone and vitamin D levels were in normal ranges (Ca = 10.2 mg/dL, *P* = 4.9mg/dL, PTH = 11.1 pg/mL, and 25hydroxy D Vitamin = 43.2 ng/mL). For the osteoporosis treatment, intravenous pamidronate therapy was initiated at 1 mg/kg/dose, three doses every four months.

**Figure 1:**
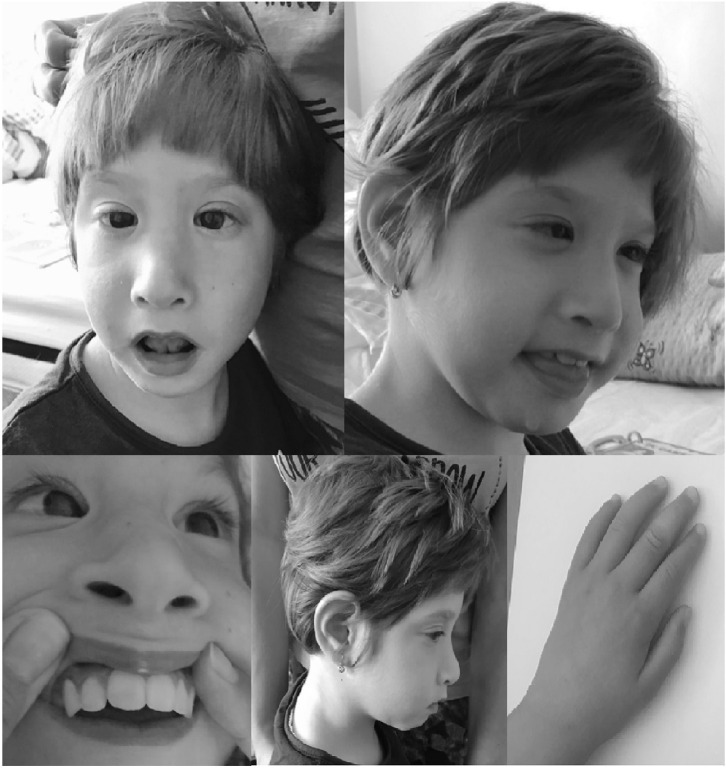
Dysmorphic features of the patient with 16q24.3 duplication (prominent anteverted ears, convergent strabismus, telecanthus, broad nasal bridge with bulbous nasal tip, long philtrum, thin upper lip, macrodontia of the upper central incisors and bilateral clinodactyly of the fifth finger). Written informed consent was obtained from the parents for publication of this case report and accompanying images.

The clinical features of the index case are presented in Figure 1.

### Genetic Testing

A G-banding karyotype of the proband was performed, which showed a double satellite in chromosome 21. Parental chromosome analysis indicated that the genetic polymorphism was paternally derived, with no associated phenotype and no significant family history of neurodevelopmental disorders or multiple congenital anomalies. The mother's karyotype was normal.

Array-based single nucleotide polymorphism (SNP array) analysis was performed using Infinium OmniExpress-24 v1.2 Kit (Illumina), Human Genome Build 37 (hg19). The interpretation was based on Genome Studio 2.0 software. All genetic investigations performed on this patient and her family members were done after informed consent was obtained following local Institutional Review Board policies and procedures.

**Figure 2:**
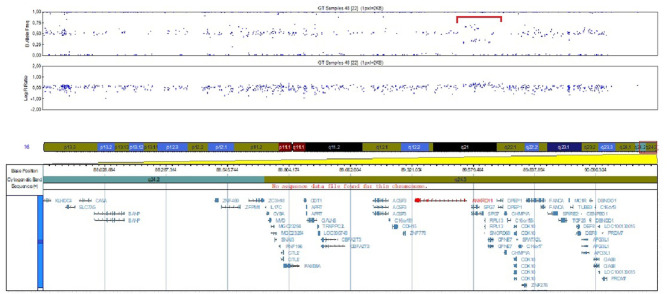
Image of the 16q24.3 duplication identified by SNP array analysis.

SNP array analysis identified arr[GRCh37]16q24.3(89542695_89656251)x3, a 113.556 bp duplication including four OMIM genes, three of them morbid OMIM genes: *ANKRD11, RPL11* and *PGN* (Figure 2). This duplication event involves the ANKRD11 gene, including the promoter, 5'UTR region and the first exon, the breaking point being in intron 1.

It was also performed exome analysis, to assess the genes possibly associated with osteoporosis, mainly osteogenesis imperfecta and, also the genes associated with neurodevelopmental disorders, no pathogenic variants, or VUS (variants of uncertain significance) were detected.

## Discussion

We presented a female patient with developmental delay, craniofacial dysmorphism, short stature, and very low bone mineral density with 16q24.3 duplication of 113 kb, involving three morbid OMIM genes, *ANKRD11, RPL13*, and *PGN*. The exome testing did not reveal pathogenic or VUS variants in genes possibly associated with this clinical picture.

*ANKRD11* gene haploinsufficiency has been reported in patients with KBG syndrome. Sporadic and familial cases have been described with an autosomal dominant inheritance pattern. The pathogenic variants reported in the literature were mainly represented by: heterozygous splice site variants, nonsense and frameshift variants, intragenic deletion, or duplication in the *ANKRD11* gene (3, 7, 11–22).

The duplication observed in our patient has not been reported until now, larger CNVs including this one has been mentioned in the ClinGen and Decipher databases, in association with developmental delay, intellectual disability or autistic spectrum disorders.

Crippa et al. reported the only cases described in literature with KBG syndrome and *ANKRD11* microduplication (of the region between exon 4 and exon 8) and these authors demonstrated the presence of two mutant *ANKRD11* transcripts containing a premature stop codon, allowing to predict a truncated non-functional protein (20). The patients described by these authors presented a moderate clinical presentation with facial dysmorphism, minor skeletal anomaly (brachymetacarpia), kidney anomalies, and mild cognitive impairment (20). In our case the duplication involved *ANKRD11* gene in his first region, including promoter, 5′UTR region and the first exon, which could have an influence on gene expression, functional studies were not performed to validate specific pathogenic relevance of this CNV.

*ANKRD11* gene code for a protein with 2663 amino acids, the C terminal region (between 2,369 and 2,663 amino acids) containing an important domain for *ANKRD11* protein degradation, which presents D-box sequences as signals for proteasome degradation (23, 24). Thus, the C terminal region play a role in regulating protein abundance, the deletions or nucleotide variants of this regions, being the main mechanism for KBG syndrome, by a dominant negative effect (24). The N-terminal part could be associated with changes protein–protein interaction and homodimer synthesis (24). This gene also has four repeated domains, ANK1, ANK2, ANK3, ANK4 (between the amino acids 167–292) which permit the interactions with other proteins, as histone deacetylases and p160 coactivators, thus controlling the genes expression (4, 23). These effects are important for neuron adaptability and plasticity, also for bone development (25, 26). It is known that *ANKRD11* expression levels are regulated during the cell cycle, with a maximal level at M phase and a rapidly descent after this phase (24).

The duplication seen in our patient, involving the promoter region, may influence gene expression regulation, which could influence the protein abundance and the exact expression during the cell cycle, thus being possible pathogenetic mechanisms for the clinical picture seen in this patient.

In Table 1 we mentioned the main cases described in the literature with CNVs at 16q24.3 region, involving the *ANKRD11* gene. The phenotypic features of these patients usually have met the diagnostic criteria (6, 7). The great majority of these CNVs are *ANKRD11* deletions, inducing haploinsufficiency for this gene.

**Table 1:** Patients described with CNV of 16q24.3 region involving *ANKRD11* gene.

**References**	**16q24.3 CNVs**	**Size**	**Position (hg19)**	**Dysmorphic features**	**Internal malformations**	**DD/ID**	**Other neuropsychiatric features**	**Short stature**	**Skeletal abnormalities**
Willemsen et al. (11)	del	378kb	89.12–89.53	Yes	CNS Skeletal Ocular	Moderate DD/ID	Seizures	p3-10	Kyphoscoliosis
Willemsen et al. (11)	del	265kb	89.27–89.53	Yes	CNS	Borderline DD/ID	Speech delay ASD	p10-25	-
Lim et al. (12)	del	240kb	89.35-89.59	Yes	Skeletal	Borderline DD/ID	Seizures, ADHD, Speech delay	<p3	Delayed bone age, narrowing and elongation of iliac wings and body, small femoral heads, small distal tibial epiphyses, and tibiotalar slanting
Isrie et al. (13)	del	221kb	89.34-89.56	Yes	Skeletal	Borderline DD/ID	ADHD, delayed speech and language development	<p3	Clinodactyly, syndactyly
Isrie et al. (13)	del	138kb	89.33-89.47	Yes	Skeletal	Borderline DD/ID	Seizures ADHD	<p3	Short 4th and 5th metacarpals
Scarano et al. (14)	del	140kb	89.28–89.42	Yes	Skeletal, renal, genital	DD/ID	Behavioral features	N	Delayed bone age, ribs abnormalities, joint stiffness, clinodactyly, short metacarpals
Scarano et al. (14)	del	220kb	89.33-89.55	Yes	Skeletal, cardiac	DD/ID	Behavioral features	<p3	Delayed bone age, ribs abnormalities, scoliosis/kyphosis, joint stiffness, clinodactyly
Scarano et al. (14)	del	130kb	89.42-89.55	Yes	Skeletal	DD/ID	Behavioral features	N	Delayed bone age, ribs abnormalities, joint stiffness, clinodactyly
Khalifa et al. (15)	del	197kb	89.38-89.58	Yes	Cardiac, genital, digestive skeletal	DD/ID	Febrile seizures	p10	Delayed bone age, small hands with brachydactyly, partial syndactyly 2nd-3rd toes
Youngs et al. (16)	Del	180kb	89.39-89.57	Yes	-	mild ID	ASD, ADHD, obsessive–compulsive disorder, anxiety, self-injury	<p3	-
Spengler et al. (17)	del	348kb	89.37-89.60	Yes	Skeletal	No	No	<p3	Clinodactyly 5th finger
Novara et al. (18)	del	340kb	89.16-89.50	Yes	Skeletal	ID	Stereotypies bruxism, hand-biting, sleep disorders	<p3	-
Sacharow et al. (19)	del	320 kb	89.28-89.60	Yes	Skeletal, cardiac, genital	ID	Speech delay	p50	Clinodactyly 5th finger
Crippa et al. (20)	dup	89 kb	89.35-89.43	Yes	Skeletal, cardiac, renal	moderate ID		<p3	Short metacarpals
Our patient	dup	113kb	89.54-89.6	Yes	Skeletal, cardiac	DD/ID	ASD, seizures, speech delay	<p3	Low bone mineral density, fracture

*Del, deletion; dup-duplication; CNV, copy number variants; DD/ID, developmental delay/intellectual disability; ADS, autism spectrum disorder; ADHD, Attention-Deficit/Hyperactivity Disorder; CNS, central nervous system; N, normal*.

At our patient, two major criteria (macrodontia, height below 10th centile) and three minor criteria (hand anomaly, seizures, formal diagnosis of autism) were fulfilled (associated to developmental and speech delay), taking into account the criteria of Low et al. (8), which permitted us to affirm the clinical diagnosis of KBG syndrome, even if the genetic testing firstly suggested this diagnosis.

*RPL13*, another gene involved in this duplication, code for a protein incorporated in the 60S subunit of ribosomes, and was very recently described by Le Caignec et al. to be associated with a human ribosomopathy, Isidor-Toutain spondyloepimetaphyseal dysplasia, an autosomal dominant disorder (27). These authors showed that *RPL13* gene is highly expressed in osteoblasts and chondrocytes from hypertrophic and remodeling zones in mouse growth plate (27). *RPL13* gene duplication is not known to be associated with human disorder, but the high *RPL13* expression in bones and the association with a skeletal dysplasia in case of heterozygous splice variant could have a role for the very low bone density seen in the present case (27). Also, it is known that *RPL13* gene is a very evolutionary conserved gene and a duplication at this level might have a certain impact, that for the moment we cannot prove. However, the neuropsychiatric picture seen in our patient is not so obvious correlated with *RPL13* gene, the patients with *RPL13* splice mutation described by Le Caignec et al. being without neuropsychiatric pathology (27).

*CPNE7* gene is not known to be associated with human pathology and *PGN* is associated with an autosomal recessive form of spastic paraplegia when two alleles are affected (28). In our patient, the CNV involved only one copy of the PGN gene. Therefore, it is less likely to be involved in the etiology of the syndrome in the given case, unless a small variant is not presented on the second allele, this hypothesis being excluded after exome sequencing. Additionally, in the absence of special neurological findings of spastic paraplegia, we considered its role less probable in the aetiologic background of the presented clinical characteristics in our index case.

A particularity of our case is the skeletal involvement as the very low bone density, which has not been clearly described in other cases with KBG syndrome where skeletal involvement is present, but mainly noticed under other phenotypes (kyphoscoliosis, delayed bone age, clinodactyly, short metacarpals, short fingers, costovertebral abnormalities, and hip dysplasia). However, experimental studies on Yoda mutant mice showed that heterozygous missense mutation in *ANKRD11* gene is associated with reduced body size, craniofacial abnormalities and reduced bone mineral density (26). This missense mutation was produced in the C terminal region, affecting a very well conserved repressive transcriptional domain, thus eliminating an inhibitory effect on gene expression, a mechanism that might destabilize the whole protein. This might be another mechanism different than the well-known ANKRD11 haploinsufficiency, responsive for the skeletal phenotype in KBG syndrome. Low mineral density, as observed in our patient has not been described until now in patients with KBG syndrome. This observation raises the question of whether the alteration of the first exon of the *ANKRD11* gene could be more important in determining bone mineral density. Functional studies were not possible to argue this hypothesis. It was also assessed another possible independent event, possibly associated with this duplication, by doing exome analysis, but no pathogenic variants or VUS in genes involved in osteogenesis imperfecta or osteoporosis and also in genes associated with developmental delay/intellectual disability, were observed. A very recent observation about *RPL13* gene and skeletal dysplasia lead to a hypothesis, which must be further evaluated, about the importance of *RPL13* duplication for the extremely low bone density seen in this patient.

Another particularity of this case report is the “genotype first” approach. The clinical phenotype being not specific enough for KBG syndrome, it also indicates other possible pathologies, such as Cornelia de Lange syndrome, fetal alcohol spectrum disorder or Cohen syndrome. Parenti et al. published some cases that demonstrated overlap between Cornelia de Lange syndrome and KBG syndrome, with common features such as intellectual disability, behavioral issues, autism spectrum disorders, and craniofacial abnormalities (microcephaly, synophrys, long eyelashes, and arched eyebrows) (29). In our patient, we observed some similarities to mild form of Cornelia de Lange syndrome, such as microcephaly, cognitive impairment, behavioral problems, autism spectrum disorder, the appearance of the eyebrows, and eyelashes and postnatal growth retardation. Fetal alcohol spectrum disorder was also suggested by some patient's features, such as: microcephaly, dysmorphic signs as long philtrum and thin upper lip, developmental delay/intellectual deficiency and growth restriction (30).

The intellectual delay observed in our case, associated with some dysmorphic signs such as prominent central incisors, microcephaly, and hypotonia, also indicated Cohen syndrome, as differential diagnosis (31). However, the somatic delay observed in our case is in contradiction to the tendency to truncal obesity, often age-dependent, seen in Cohen syndrome.

The exome testing did not show pathogenic or VUS variant in the genes involved in Cornelia de Lange syndrome or Cohen syndrome.

In this patient, the birth was described as complicated, relevant to perinatal ischaemia, which could be a cause for neurologic issues, but the presence of associated dysmorphism and bicuspid aortic valve make this hypothesis less probable.

## Conclusions

This 16q24.3 duplication and the clinical picture of the patient, which could be concordant with KBG syndrome phenotype, led to CNV interpretation as variant of uncertain significance (VOUS) but with arguments for potential pathogenicity which have to be proven, also other potential contributing causes cannot be excluded and functional studies are needed.

To our knowledge, this case is a first genetically confirmed Romanian patient with microduplication involving *ANKRD11*. The patient's presented a subtle clinical picture of KBG syndrome, diagnosis firstly suggested by SNP array analysis. The particularity of the case was the presence of a very low bone mineral density in a patient with 16q24.3 microduplication. *ANKRD11* gene haploinsufficiency is known to be associated with skeletal involvement, usually involving various malformations, short stature or delayed bone age, with an effect on bone mass observed only in experimental studies on mice with induced mutations on the *ANKRD11* gene.

## Data Availability Statement

The datasets presented in this article are not readily available because their exhaustiveness, the original contributions presented in the study are included in the article. Requests to access the datasets should be directed to Miclea Diana, diana.miclea@umfcluj.ro.

## Ethics Statement

The studies involving human participants were reviewed and approved by Ethics Committee of Iuliu Hatieganu University of Medicine and Pharmacy. Written informed consent to participate in this study was provided by the participants' legal guardian/next of kin. Written informed consent was obtained from the individual(s), and minor(s)' legal guardian/next of kin, for the publication of any potentially identifiable images or data included in this article.

## Author Contributions

SB, DM, and PG-S contributed to conceptualisation, methodology, validation, investigation, manuscript writing, and manuscript supervising. CL, CA, and AK contributed to manuscript writing and methodology of the study. All authors contributed to the article and approved the submitted version.

## Conflict of Interest

The authors declare that the research was conducted in the absence of any commercial or financial relationships that could be construed as a potential conflict of interest.
